# Supplementary material for: A comparative assessment of adult mosquito trapping methods to estimate spatial patterns of abundance and community composition in southern Africa
Source: Parasit Vectors. 2019 Oct 2;12:462. doi: 10.1186/s13071-019-3733-z (PMC6775653; doi:10.1186/s13071-019-3733-z)
Supplement: Supplementary file 1 — Additional file 1: Table S1. The number of females collected by trap and site. The mosquito column (mosq) indicates the number of females collected; the traps column of trapping nights represented. Table S2. The number of females collected of each species by region of the park. Table S3. Summary of weather conditions sampled within each region of the park. Numbers are displayed as median and range in parentheses. [file 13071_2019_3733_MOESM1_ESM.pdf]

Additional file 1

Additional descriptive data tables

Mosquito data

The data capture 200 total trap-nights and resulted in 40 BG-CDC comparisons, 40 BG-GAT comparisons, 40 BG-net comparisons, 50 CDC-GAT comparisons, and 50 CDC-net comparisons. Below, we provide summary statistics for the mosquito data aggregated across 3-4 sampling nights at each site (Table S1) and aggregated across sampling nights for each species (Table S3). Sites vary in trapping effort due to rainfall in Satara and Punda Maria as well as animal interference. This aggregated data is displayed for summary purposes, as analyses of mosquito abundance compare the number of mosquitoes collected per night between functioning traps.

Weather data

Regions of the park were characterized by distinct weather patterns, as temperature was significantly different between regions (ANOVA: temperature, $F_{(3, 10)} = 4.88$, $P<0.024$; wind speed, $F_{(3, 10)} = 0.41$, $P = 0.752$; relative humidity, $F_{(3, 10)} = 1.97$, $P = 0.183$).

Table S1. The number of females collected by trap and site. The mosquito column (mosq) indicates the number of females collected; the traps column of trapping nights represented.

Trap	BG		CDC		GAT		net		Notes
	mosq	traps	mosq	traps	mosq	traps	mosq	traps	
Malelane Total	14	14	150	16	4	15	159	15	(total - across 4 nights)
Malelane 1	5	4	79	4	0	4	19	4	
Malelane 2	4	3	38	4	0	4	72	4	
Malelane 3	1	3	15	4	0	3	20	4	
Malelane 4	4	4	18	4	4	4	48	3[a]	a. Net not set-up, elephants
Satara Total	12	9	175	11	1	12	80	11	(total - across 3 nights)
Satara 1	9	3	26	3	0	3	24	3	
Satara 2	1	2	15	3	1	3	23	3	
Satara 3	0	1[b]	128	3	0	3	23	2	b. Two BG traps destroyed, hyena
Satara 4	0	3	6	2[c]	0	3	10	3	c. CDC pulled down, baboons
Shingwedzi Total	3	10	51	15	0	15	146	15	(total - across 4 nights)
Shingwedzi 1	2	3	0	3	0	3	21	3	
Shingwedzi 2	0	2[d]	11	4	0	4	9	4	d. BG trap not deployed, previous damage
Shingwedzi 3	1	3	17	4	0	4	93	4	
Shingwedzi 4	0	2[c,d]	23	4	0	4	23	4	d. BG trap not deployed, previous damage<br>b. One BG trap destroyed, hyena
Punda Maria Total	1	8	18	11	0	12	62	11	(total - across 3 nights)
Punda Maria 1	1	3	1	3	0	3	12	3	
Punda Maria 2	0	2[d]	0	2	0	3	31	3	d. BG trap not deployed, previous damage
Punda Maria 3	0	2[d]	7	3	0	3	11	3	d. BG trap not deployed, previous damage
Punda Maria 4	0	1[d]	10	3	0	3	8	2[e]	d. BG trap not deployed, previous damage<br>e. tent trap fell over, unknown animal
**Overall Total**	30	41	349	53	5	54	447	52	

Table S2. The number of females collected of each species by region of the park.

Species	Malelane	Skukuza	Satara	Shingwedzi	Punda Maria	Total
Aedes aegypti	25	0	4	0	0	29
Aedes aerarius	2	0	0	0	0	2
Aedes dentatus complex	4	0	1	0	1	6
Aedes mcintoshi	1	0	11	0	0	12
Aedes metallicus	0	0	1	1	0	2
Aedes ochraceus	0	0	19	8	1	28
Aedes quasiunivittatus	5	0	1	0	0	6
Aedes sudanensis	3	1	15	2	0	21
Aedes unidentatus	3	0	0	0	0	3
Aedes vexans complex	18	1	120	9	0	148
Aedes vittatus	3	0	0	1	0	4
Anopheles coustani	9	4	5	0	1	19
Anopheles funestus	15	0	2	0	1	18
Anopheles gambiae s.l.	23	0	12	32	9	76
Anopheles maculipalpis	0	0	0	3	0	3
Anopheles pretoriensis	28	1	4	24	3	60
Anopheles rufipes	8	0	1	0	2	11
Anopheles squamosus	8	0	5	1	1	15
Anopheles ziemanni	0	0	0	1	0	1
Culex antennatus	0	0	0	1	0	1
Culex bitaenorhynchus	0	0	0	1	0	1
Culex duttoni	0	0	4	3	1	8
Culex ethiopicus	17	5	2	11	1	36
Culex nebulosus	0	0	0	1	0	1
Culex pipiens complex	55	0	45	5	0	105
Culex poicilipes	3	11	1	4	2	21
Culex simpsoni	4	0	0	2	0	6
Culex theileri	2	0	1	4	77	84
Culex trifoliatus	33	2	0	0	0	35
Culex univittatus complex	50	10	22	79	9	170
Lutzia tigripes	0	0	0	0	1	1
Mansonia africana	1	3	0	2	0	6
Mansonia uniformis	0	0	2	3	1	6
Uranotaenia balfouri	0	0	0	1	0	1
3 unidentified *Anopheles* species	2/1/3	0/0/0	0/0/0	0/0/0	0/0/0	2/1/3
Unidentified *Culex* species	1	0	0	0	0	1
Unidentified *Mimomyia* species	0	0	1	0	0	1
Unidentified Genus	0	0	0	1	0	1

Table S3. Summary of weather conditions sampled within each region of the park. Numbers are displayed as median and range in parentheses.

	temperature	wind speed	relative humidity
Malelane	22.9 (21.2-25.1)	0.33 (0-0.75)	83.8 (60.7-93.7)
Satara	22.0 (21.7-25.6)	0.23 (0-0.68)	73.3 (73.3-84.0)
Shingwedzi	18.3 (15.1-21.7)	0.10 (0-0.38)	90.1 (86.4-91.8)
Punda Maria	16.6 (16.5-20.4)	0.00 (0-0.55)	71.0 (62.6-84.7)
