# Supplementary material for: A comparative assessment of adult mosquito trapping methods to estimate spatial patterns of abundance and community composition in southern Africa
Source: Parasit Vectors. 2019 Oct 2;12:462. doi: 10.1186/s13071-019-3733-z (PMC6775653; doi:10.1186/s13071-019-3733-z)
Supplement: Supplementary file 2 — Additional file 2: Text S1. Additional methods and results for the regression analysis. Table S4. Model parameters, estimates, standard error (SE) and hypothesis tests for the Poisson regression analyses in Fig. 3. [file 13071_2019_3733_MOESM2_ESM.pdf]

Additional file 2

Text S1. Additional methods and results for the regression analysis

All models consider the counts of mosquito from each night i with the response variable, μ_i. Explanatory variables include a factor variable for the effect of the region that trapping occurred in ($region_k$) and continuous variables for the effect of wind speed, temperature and relative humidity ($windspeed_i$, $temperature_i, RH_i$). We additionally included a site-specific intercept, $\propto_{j[i]}$, with subscript notation to indicate which of the j sites were sampled in night i. This results in the following regression model,

$$\ln(\mu_i) = \propto_{j[i]} + \beta_{1,k} region_k + \beta_2 windspeed_i + \beta_3 temperature_i + \beta_4 RH_i + \varepsilon_i$$

where ε_i is the Poisson distributed random error.

Following model selection, we evaluate $\beta_{1,k}$ to determine how the average nightly counts of mosquitoes in region k differed from counts in Malelane, and we evaluate $\beta_2, \beta_3, \beta_4$ to determine how the average counts of mosquitoes vary with wind speed, temperature, and relative humidity, respectively. If the relative values of $\beta_{1,k}$ are consistent among traps (e.g. $\beta_{1,1} > \beta_{1,2} > \beta_{1,3}$) for models fit to data from different traps, then we conclude that trap choice does not influence spatial comparisons among regions. We were additionally interested in the variance components, as high variances indicate that counts vary across sites. We did not estimate variation due to trap position due to the number of damaged traps. However, regression models fit with the additional random effect to data from a subset of sites with full trap data resulted in similar estimates.

Table S4. Model parameters, estimates, standard error (SE) and hypothesis tests for the Poisson regression analyses in Fig. 3.

**Model**	**Estimate**	**SE**	**Z value**	**P**
**BG data** (n = 41; variance across sites = 0.062)				
$\beta_{1,1}$ – Satara vs. Malelane	0.280	0.449	0.623	0.533
$\beta_{1,2}$ – Shingwedzi vs. Malelane	-0.073	0.828	-0.089	0.930
$\beta_{1,3}$ – Punda Maria vs. Malelane	-0.935	1.183	-0.791	0.429
β_3 – temperature	0.700	0.330	2.117	0.034
**CDC data** (n = 52; variance across sites = 0.621)				
$\beta_{1,1}$ – Satara vs. Malelane	0.049	0.579	0.085	0.933
$\beta_{1,2}$ – Shingwedzi vs. Malelane	-0.508	0.613	-0.829	0.407
$\beta_{1,3}$ – Punda Maria vs. Malelane	-1.215	0.657	-1.850	0.064
β_2 – wind speed	-0.098	0.059	-1.653	0.098
β_3 – temperature	0.498	0.102	4.864	<0.001
**Net data** (n = 52; variance across sites = 0.326)				
$\beta_{1,1}$ – Satara vs. Malelane	-0.388	0.424	-0.916	0.359
$\beta_{1,2}$ – Shingwedzi vs. Malelane	0.338	0.445	0.759	0.448
$\beta_{1,3}$ – Punda Maria vs. Malelane	-0.522	0.443	-1.179	0.238
β_2 – wind speed	-0.348	0.063	-5.545	<0.001
β_3 – temperature	0.344	0.079	4.336	<0.001
β_4 – relative humidity	-0.271	0.071	-3.823	0.001
**Net + CDC data** (n = 50, variance across sites = 0.181)				
$\beta_{1,1}$ – Satara vs. Malelane	-0.385	0.330	-0.742	0.458
$\beta_{1,2}$ – Shingwedzi vs. Malelane	-0.245	0.330	-0.724	0.458
$\beta_{1,3}$ – Punda Maria vs. Malelane	-0.651	0.340	-1.916	0.055
β_2 – wind speed	-0.217	0.044	-4.909	<0.001
β_3 – temperature	0.298	0.062	4.782	<0.001
**All data combined** (n = 38, variance across sites = 0.222)				
$\beta_{1,1}$ – Satara vs. Malelane	-0.288	0.382	0.752	0.452
$\beta_{1,2}$ – Shingwedzi vs. Malelane	-0.148	0.382	-0.387	0.699
$\beta_{1,3}$ – Punda Maria vs. Malelane	-0.688	0.404	-1.704	0.088
β_2 – wind speed	-0.107	0.051	-2.095	0.036
β_3 – temperature	0.432	0.076	5.702	<0.001
