# Supplementary material for: A comparative assessment of adult mosquito trapping methods to estimate spatial patterns of abundance and community composition in southern Africa
Source: Parasit Vectors. 2019 Oct 2;12:462. doi: 10.1186/s13071-019-3733-z (PMC6775653; doi:10.1186/s13071-019-3733-z)
Supplement: Supplementary file 4 — Additional file 4: Table S6. Descriptive results comparing species-specific shifts in mosquito communities collected in the net and CDC trap. Species more commonly collected in a trap are listed if 5 more were collected in that trap after all sampling days at the site. Figure S1. The apparent richness (number of unique species) and diversity for sites within each region. Figure S2. Non-metric multidimensional scaling ordinations of trap differences in mosquito communities in Malelane, Satara, Shingwedzi and Punda Maria. Figure S3. Species-specific trap preferences for the net vs CDC trap difference based on rare species not displayed in Fig. 3. Dots represent the difference in the number of mosquitoes collected in the net vs the CDC trap based on the total number of mosquitoes sampled across nights at each site. Figure S4. The net trap and the CDC trap caught higher numbers of mosquitoes (Fig. 3) and this pattern was not driven by any species or genus-specific trap bias (left figures) but by variation in the total number of the species collected (right figures). Figure S5. Dendrogram of species composition based on Bray-Curtis dissimilarity and the hierarchical clustering algorithm. [file 13071_2019_3733_MOESM4_ESM.pdf]

Additional file 4

Additional results

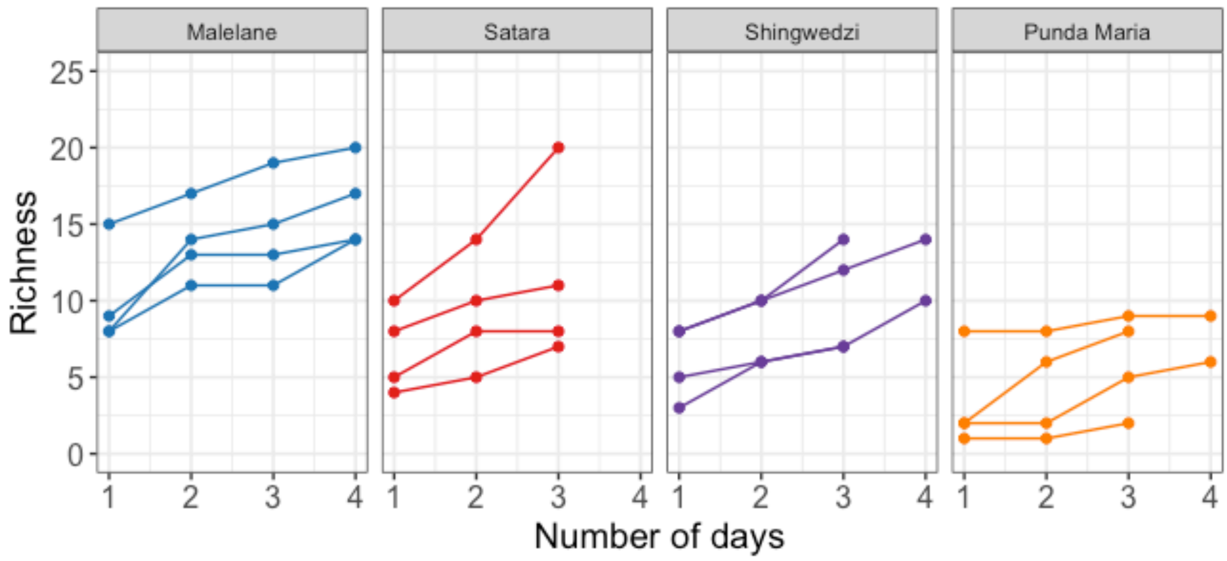

Figure S1. The apparent richness (number of unique species) and diversity for sites within each region.

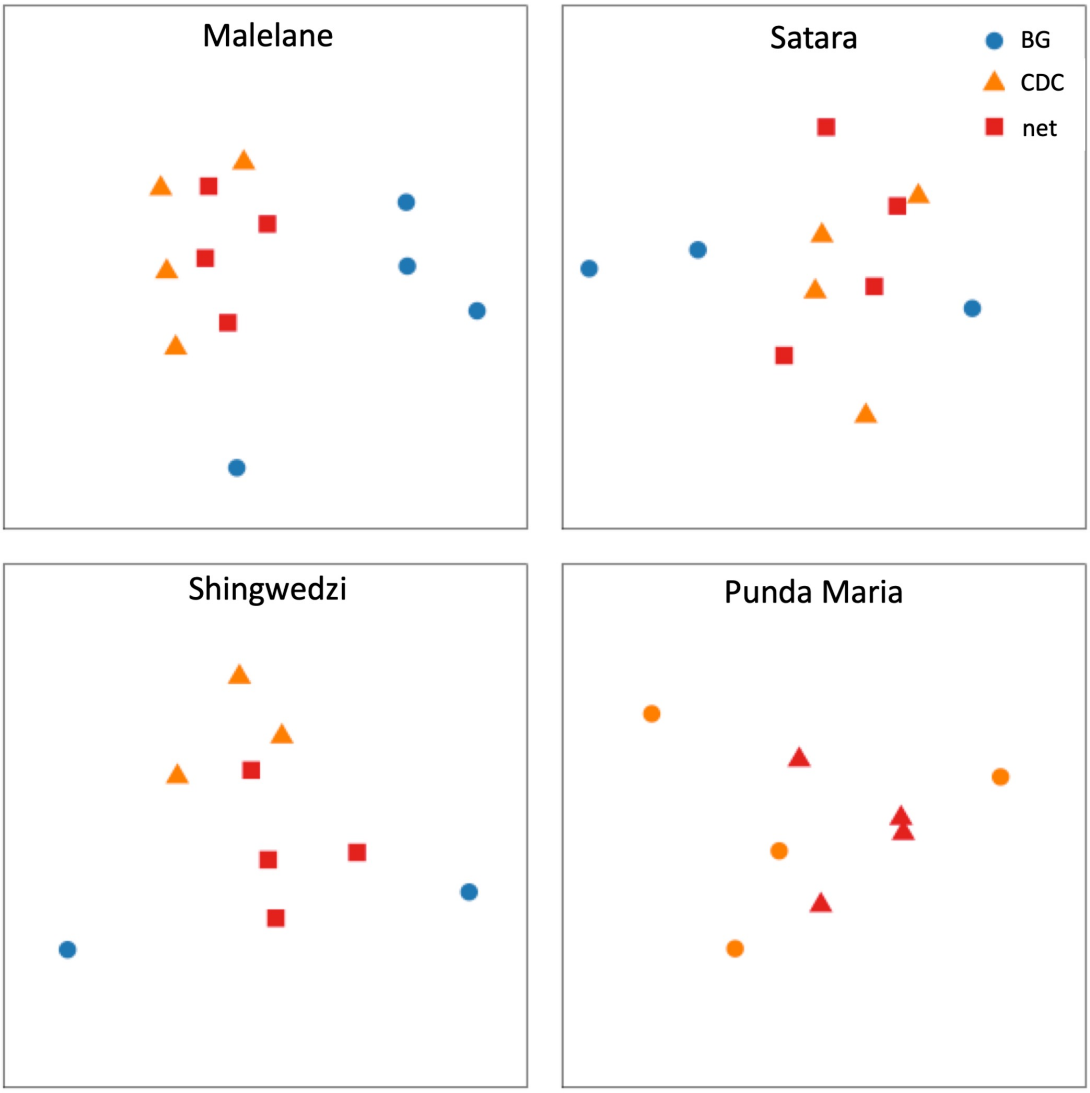

Figure S2. Non-metric multidimensional scaling ordinations of trap differences in mosquito communities in Malelane, Satara, Shingwedzi and Punda Maria.

Table S6. Descriptive results comparing species-specific shifts in mosquito communities collected in the net and CDC trap. Species more commonly collected in a trap are listed if 5 more were collected in that trap after all sampling days at the site.

Site	Species common to the CDC trap	Species common to the net trap
Malelane 1	*C. univittatus* complex*; C. trifoliatus; C. pipiens* complex*; Ae. vexans* complex	-
Malelane 2	*C. univittatus* complex	*An. gambiae s.l.; An. pretoriensis; C. trifoliatus; Ae. vexans* complex
Malelane 3	-	-
Malelane 4	-	*C. pipiens* complex
Satara 1	*C. pipiens* complex	*Ae. vexans* complex
Satara 2	-	*C. pipiens* complex
Satara 3	*Ae. vexans* complex; *An. gambiae s.l.; An. squamosus*	-
Satara 4	-	-
Shingwedzi 1	-	*C. univittatus* complex
Shingwedzi 2	-	-
Shingwedzi 3	-	*C. univittatus* complex*; An. gambiae* complex*; An. pretoriensis*
Shingwedzi 4	*Ae. vexans* complex	-
Punda Maria 1	-	*C. theileri*
Punda Maria 2	-	*C. theileri*
Punda Maria 3	-	-
Punda Maria 4	*C. theileri*	-

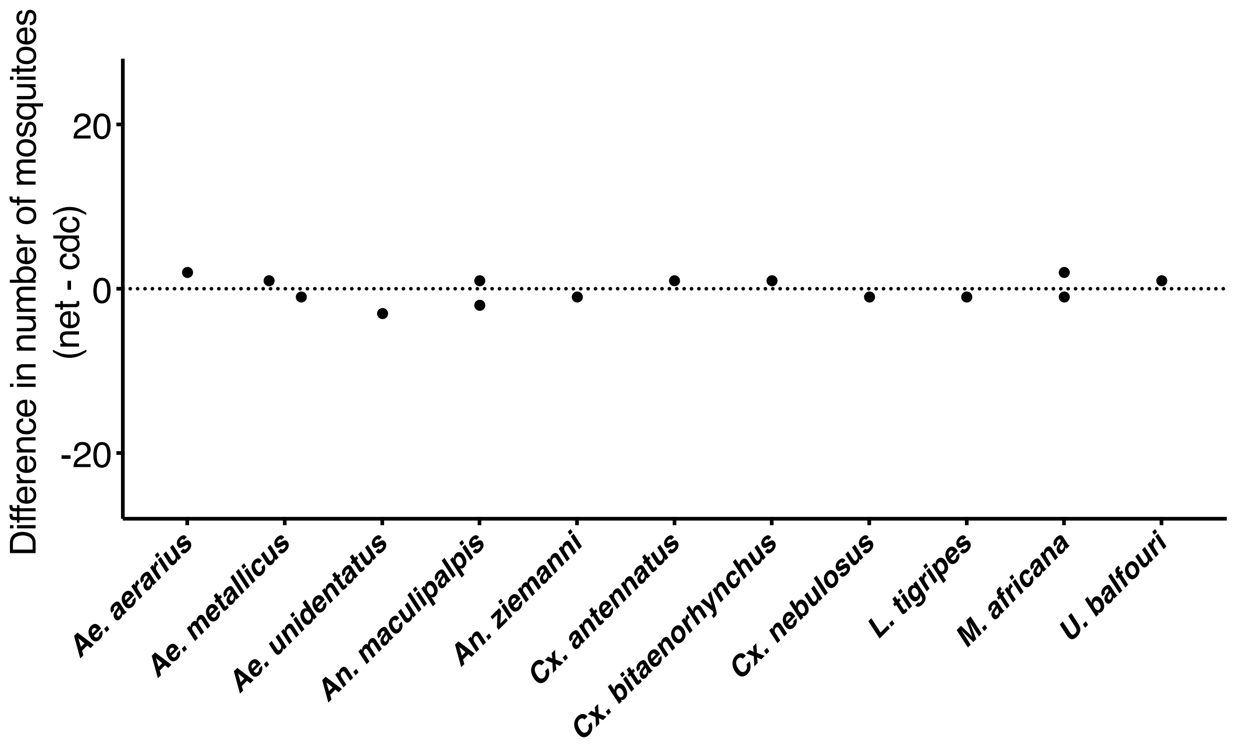

Figure S3. Species-specific trap preferences for the net *vs* CDC trap difference based on rare species not displayed in Fig. 3. Dots represent the difference in the number of mosquitoes collected in the net *vs* the CDC trap based on the total number of mosquitoes sampled across nights at each site.

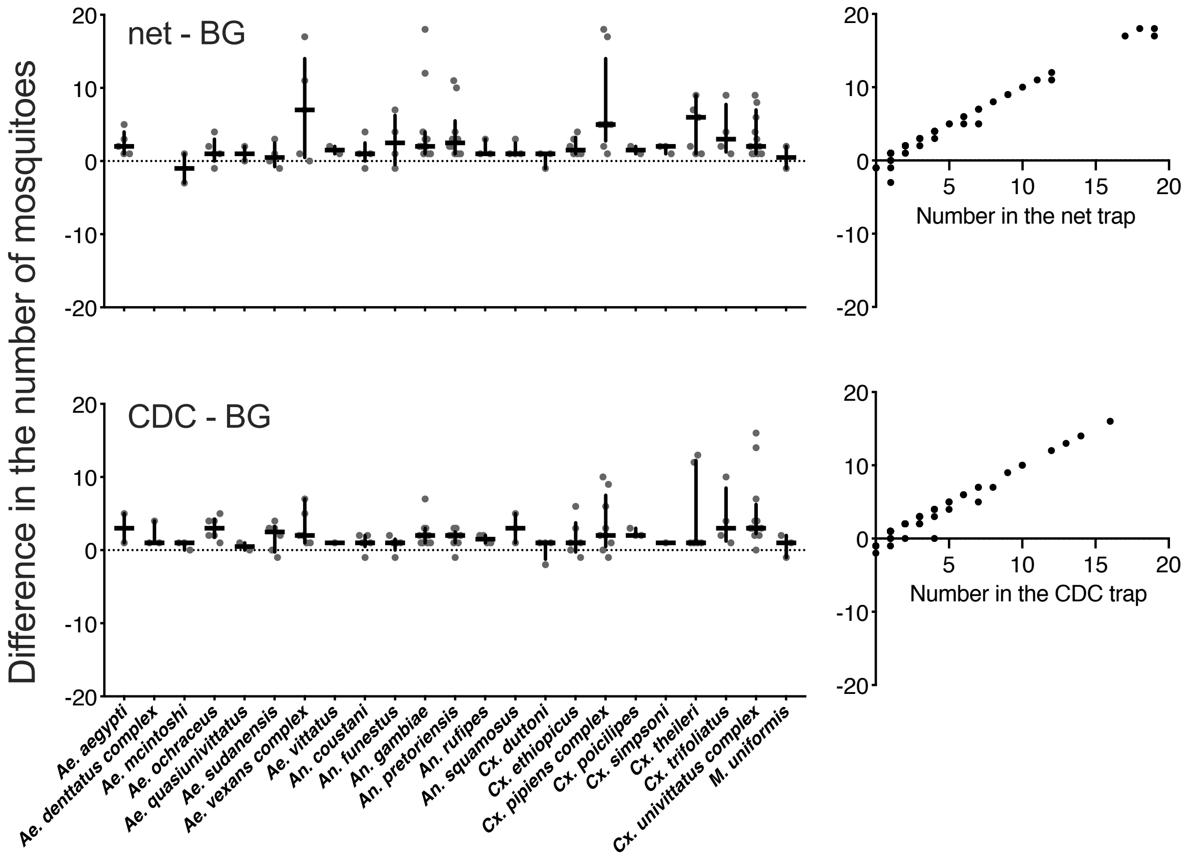

Figure S4. The net trap and the CDC trap caught higher numbers of mosquitoes (Fig. 3) and this pattern was not driven by any species or genus-specific trap bias (left figures) but by variation in the total number of the species collected (right figures).

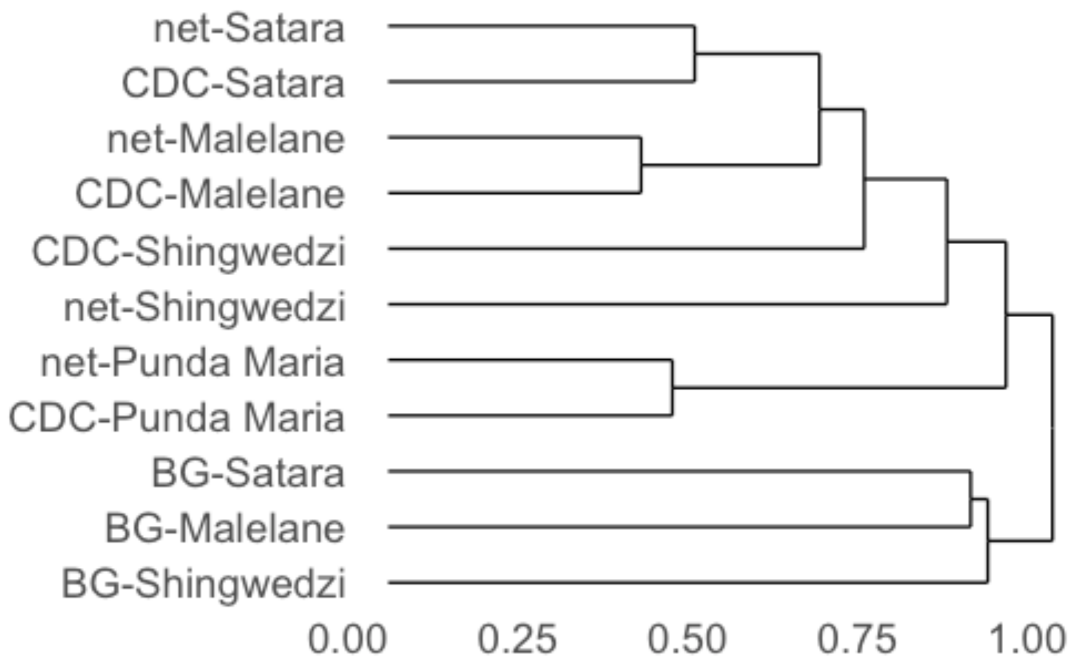

Figure S5. Dendrogram of species composition based on Bray-Curtis dissimilarity and the hierarchical clustering algorithm.
